# Growth hormone in fertility and infertility: Mechanisms of action and clinical applications

**DOI:** 10.3389/fendo.2022.1040503

**Published:** 2022-11-14

**Authors:** Chia-Wei Chang, Yu-Wen Sung, Ya-Wen Hsueh, Yi-Yan Chen, Ming Ho, Hsi-Chen Hsu, Tung-Chuan Yang, Wu-Chou Lin, Hsun-Ming Chang

**Affiliations:** Department of Obstetrics and Gynecology, China Medical University Hospital, Taichung, Taiwan

**Keywords:** growth hormone, insulin-like growth factor 1, ovarian function, endometrial receptivity, infertility management, poor ovarian response, recurrent implantation failure, oocyte donation

## Abstract

Secreted by the anterior pituitary gland, growth hormone (GH) is a peptide that plays a critical role in regulating cell growth, development, and metabolism in multiple targeted tissues. Studies have shown that GH and its functional receptor are also expressed in the female reproductive system, including the ovaries and uterus. The experimental data suggest putative roles for GH and insulin-like growth factor 1 (IGF-1, induced by GH activity) signaling in the direct control of multiple reproductive functions, including activation of primordial follicles, folliculogenesis, ovarian steroidogenesis, oocyte maturation, and embryo implantation. In addition, GH enhances granulosa cell responsiveness to gonadotropin by upregulating the expression of gonadotropin receptors (follicle-stimulating hormone receptor and luteinizing hormone receptor), indicating crosstalk between this ovarian regulator and the endocrine signaling system. Notably, natural gene mutation of GH and the age-related decline in GH levels may have a detrimental effect on female reproductive function, leading to several reproductive pathologies, such as diminished ovarian reserve, poor ovarian response during assisted reproductive technology (ART), and implantation failure. Association studies using clinical samples showed that mature GH peptide is present in human follicular fluid, and the concentration of GH in this fluid is positively correlated with oocyte quality and the subsequent embryo morphology and cleavage rate. Furthermore, the results obtained from animal experiments and human samples indicate that supplementation with GH in the *in vitro* culture system increases steroid hormone production, prevents cell apoptosis, and enhances oocyte maturation and embryo quality. The uterine endometrium is another GH target site, as GH promotes endometrial receptivity and pregnancy by facilitating the implantation process, and the targeted depletion of GH receptors in mice results in fewer uterine implantation sites. Although still controversial, the administration of GH during ovarian stimulation alleviates age-related decreases in ART efficiency, including the number of oocytes retrieved, fertilization rate, embryo quality, implantation rate, pregnancy rate, and live birth rate, especially in patients with poor ovarian response and recurrent implantation failure.

## Introduction

In humans, the locus of the growth hormone (*GH*) gene is located in the long arm of chromosome 17, encompasses approximately 50 kb and harbors five genes: GH normal (*GH-N* or *GH1*), GH variant (*GH-V* or *GH2*), chorionic somatomammotropin hormone 1 (*CSH1*), chorionic somatomammotropin hormone 2 (*CSH2*), and chorionic somatomammotropin hormone-like 1 (*CSL*) (1). GH is an essential hormone for optimal female reproduction, as decreased fertility has been reported in women with GH deficiency. Additionally, GH replacement can enable successful pregnancy in these infertile women (2, 3). Targeted depletion of GH receptor (*GHR*) in mice resulted in a decrease in various fertility parameters similarly seen in GH- deficient women (4). In the agriculture industry, injection with GH associated with ovulation induction or at the time of insemination enhanced the pregnancy rate in cattle (5, 6). In the past three decades, the functional role of GH in regulating female reproduction has attracted increasing attention and has become a focus of reproductive and endocrine research. Several publications have revealed that locally produced GH and its downstream signaling are implicated in the function of mammalian ovaries and that they participate in the regulation of various follicular functions (7). In clinical practice, GH has widely been applied as an adjuvant reagent to promote various clinical outcomes in different scenarios during assisted reproductive technology (ART), aiming to increase collected oocyte numbers, improve oocyte/embryo quality, and improve pregnancy and live birth rates (8–10). A comprehensive understanding of the expression, actions and underlying molecular mechanisms of the GH/GHR system in the human ovary is critical to the development of diagnostic and/or therapeutic strategies for women suffering from infertility and ovulation disorders. In this review, we also summarize the scientific literature related to the clinical applications of recombinant human GH in ART.

## Growth hormone in the female reproductive system

### Pituitary and extrapituitary growth hormone expression

GH-N is predominantly expressed in the pituitary gland and is upregulated by binding a transcriptional activation protein to the promoter (cis-active DNA sequences) of the *GH1* gene (11). The transcriptional activation protein (named PIT-1) belongs to the POU family of transcription factors and is found in the nuclei of somatotrophs, lactotrophs, and thyrotrophs of the pituitary (11). PIT-1 promotes the differentiation of various pituitary cell types responsible for pituitary development and growth hormone production in mammals (12). Natural mutations in the *PIT-1* gene and *GH1* gene have been identified in children with extreme growth failure (13, 14). The pituitary somatotroph is the principal site that produces circulating GH because serum levels of GH are barely detectable after hypophysectomy or pituitary ablation (15). Indeed, pituitary GH is primarily responsible for endocrine actions during perinatal and postnatal growth. In addition to the pituitary gland, GH is also expressed in multiple tissues, including the neural, ocular, immune, cardiovascular, muscular, dermal, skeletal and reproductive systems in humans (16). In these extrapituitary tissues, the locally produced GH acts to regulate the proliferation, differentiation and metabolism of the adjacent cells in an autocrine or paracrine manner even prior to the ontogeny of the pituitary gland (16). Recent studies have demonstrated the significant roles of extrapituitary GH in physiology, pathophysiology, and oncogenesis (17).

### Expression of growth hormone in the ovaries and uterus

All of the transcripts of the five *GH* genes (*GH1*, *GH2*, *CSH1*, *CSH2*, and *CSL*) can be detected in the ovaries of pre- and postmenopausal women (18). Using an immunofluorometric assay, a previous study showed that the corresponding proteins (GH-N, PL-A, and PL-B) of *GH1*, *CSH1*, and *CSH2* are present in the ovarian extracts of pre- and postmenopausal women (18). Additionally, the concentrations of GH-N protein are similar in pre- and postmenopausal ovaries, whereas the concentrations of CSH protein are much lower in postmenopausal ovaries (18). In female fetuses and adults, the mRNA transcripts and proteins of *GH* are detected in oocytes, stromal cells, and granulosa cells (19). GH-N plays a role in regulating some critical intraovarian functions, such as ovarian folliculogenesis, indicating that GH may act as an autocrine/paracrine factor (20).

GH has been detected in the human endometrium, as a previous immunohistochemical study showed that GH proteins are present in the glandular cells of the endometrial decidual tissues obtained from women during their late luteal phase throughout pregnancy (20). However, although intense staining for GH was identified in the glandular cells of the decidua, GH staining in the stromal cells was negative (20). A study using clinical samples demonstrated that the expression of GH in uterine epithelial cells is increased 3.4-fold in women with endometriosis and 3.8-fold in women with adenocarcinoma compared to normal women (21). In this regard, GH has been shown to not only promote cell proliferation but also suppress cell−cell adhesion. Thus elevated GH may allow endometrial cells to break away from their original sites. These results indicate that GH may be involved in the progression and pathogenesis of these diseases.

### Growth hormone receptors in the ovaries and uterus

Animal studies have shown that the GHR is expressed in the oocytes and granulosa cells of preantral follicles obtained from various species, including rats, pigs, and cattle (21–23). In humans, GHR is detected in oocytes, granulosa cells (GCs), and stromal cells of fetal and adult ovaries (19). The mRNA and protein of *GHR* are also expressed in luteal cells from a variety of mammals, including rats, pigs, cattle, and humans (24–27), indicating a functional role of GH in regulating luteal function.

The uterus is another target site of GH because *Ghr* mRNA has been identified in the endometrium, glands, stroma and myometrium of the rat uterus (28). Similarly, *Ghr* mRNA has also been detected in the uterine cells of cattle, cows, and pigs (27, 29, 30). In humans, GHR is expressed in the myometrium and decidua of the uterus (17, 31). Additionally, *GHR* mRNA transcripts are detected in leiomyoma and its surrounding myometrium obtained from premenopausal women (32).

### Effects of growth hormone in the ovaries and uterus

GH is believed to enhance fertility by acting at the ovaries and uterus because *in vivo* or *in vitro* administration of GH optimizes the successful outcomes of fertility in agricultural and clinical settings (7). Additionally, GH acts as an antioxidant to reduce injury-induced tissue damage and thus improve overall ovarian health (33). Folliculogenesis and oogenesis are two critical processes required for normal fertility, and these processes are intricately regulated by the endocrine, paracrine, autocrine, and juxtacrine (via gap junctions) systems (34, 35). In this regard, GH (both pituitary and ovarian origin) plays an essential role in modulating the signals participating in this complex interplay. Locally produced GH can bind GHRs in the endoplasmic reticulum to form a GH : GHR complex, which further triggers recruitment and autophosphorylation of JAK2 (Janus kinase) at the cytoplasmic domain of GHR (36). Subsequently, the GHR/JAK2 complex induces the phosphorylation of signal transducer and activator of transcription (STAT) molecules (including STAT5a, STAT5b, STAT1, and STAT3), which translocate to the nucleus and modify gene expression and cellular activities, including cell proliferation (37).

Upon binding of gonadotropins (FSH and LH) with their corresponding gonadotropin receptors (FSHR and LHR), the downstream signaling (cyclic adenosine monophosphate/protein kinase A or cAMP/PKA) pathway induces a series of critical intrafollicular activities, including steroidogenesis, proliferation, and differentiation (38). In cultured rat granulosa cells, GH enhances FSH-induced cell differentiation by upregulating the expression of FSH receptors (39). Additionally, the combined effect of GH and FSH-induced cyclic AMP signaling mediates the upregulation of LH receptor expression and an increase in progesterone synthesis in rat granulosa cells (40). Results obtained from clinical samples showed that supplementation with GH in women with old age or decreased ovarian reserve during their *in vitro* fertilization (IVF) treatment upregulates the expression of LHR, FSHR, and GHR in human granulosa cells isolated from the collected follicular fluid samples (8, 41). All these results indicate that GH may potentiate the sensitivity and responsiveness of granulosa cells to gonadotropin or GH stimulation and subsequently regulate sex steroid synthesis and follicular development (42). The convergence of gonadotropin and GH/GHR signaling reveals crosstalk between this ovarian regulator and the endocrine signaling system.

Emerging evidence suggests that GH is an intraovarian regulator of steroidogenesis, folliculogenesis, oocyte maturation, ovulation rate, and luteal function (7). Although pituitary-derived gonadotropins are the predominant modulators of ovarian steroidogenesis, the results obtained from *in vitro* studies indicate that GH can also regulate the production of estradiol and progesterone (43, 44). In bovine granulosa cells and luteinized human granulosa cells, GH enhances the production of estradiol and progesterone (7, 45). The effects of GH on ovarian steroidogenesis in different species may vary throughout the ovary, as GH promotes basal progesterone production in porcine corpus luteum but not in developing follicles (46). In contrast, GH enhances leptin-induced progesterone production in developing follicles (47). In rat granulosa cells, GH enhances FSH-induced (but not basal) ovarian steroidogenesis, and this effect is mediated by the antagonization of BMP signaling (48). Moreover, independent of IGF-1 production and cAMP signaling, GH can promote the synthesis of androsterone and androgen in rat theca cells (49).

Animal studies have revealed the proliferative and antiapoptotic effects of GH on ovarian follicles, as administration of GH increases follicular size, follicular number, and ovarian weight (20, 50). During early folliculogenesis, the activation of primordial follicles and the subsequent development into preantral follicles are not gonadotropin-dependent, and this process is regulated by a host of locally-produced growth factors in a paracrine/autocrine manner (51). GH is likely involved in the process of activation of primordial follicles, since targeted depletion of *Ghr* in mice results in more primordial follicles and fewer primary, secondary, preantral, and antral follicles, which are associated with increased follicular atresia (52, 53). As a result, these GH-depleted animals have a lower ovulation rate, lower implantation rate, fewer corpus lutea, and small litter sizes (53). Interestingly, supplementation with exogenous GH improves the number of developing follicles in various GH-depleted mammals, including mice, buffalo, and sheep (54–56). In this regard, GH plays a critical role in stimulating the development of follicles into the gonadotropin-dependent stage. Moreover, GH may also be required for late folliculogenesis, as the absence of GH action (GHR deficiency) results in complete suppression of the development of the dominant follicle in cattle (57).

The oocyte must undergo a series of maturational processes (nuclear, epigenetic, and cytoplasmic maturation) essential for the transition from a gamete to an embryo (58). Nuclear maturation encompasses sequential events (germinal vesicle breakdown, resumption of meiosis, and release of the first polar body), which reverse meiotic arrest at prophase I and drive the progression of meiosis to metaphase II (59). Cytoplasmic maturation represents the processes (organelle redistribution, cytoskeleton dynamics, and molecular maturation) that prepare the oocyte for activation and preimplantation development (59). Of significant interest is the beneficial effect of GH on nuclear maturation and thus oocyte quality. Animal studies have demonstrated that *in vitro* administration of GH accelerated the processes of nuclear maturation in cumulus-oocyte complexes of various species, including equine, canine, bovine, and ovine species (60–63). Previous studies postulated that GH may enhance nuclear maturation by promoting cumulus cell expansion (an indicator of oocyte quality) and downregulating connexin 43 (a gap junction protein) expression. For instance, GH promotes cumulus cell expansion by stimulating cell proliferation, inhibiting apoptosis, and suppressing the synthesis of connexin 43 in canine and bovine follicles (64, 65). In addition to the beneficial effect on nuclear maturation, GH has also been shown to promote cytoplasmic maturation, as the addition of GH to the *in vitro* culture system significantly increases the cortical distribution of equine and bovine oocytes (66, 67).

Another effect of GH on ovarian follicles is its impact on the number of oocytes released in polyovulatory species, as the ovulation rate and the subsequent litter size are increased in GH-transgenic mice but reduced in GHR-KO mice (68, 69). Additionally, pretreatment with GH during superovulation regimens increases the number of retrieved oocytes in sheep (56). In ewes, cotreatment with GH and FSH during superovulation induction also increases the number of transferable embryos because of a decreased number of unfertilized eggs and degenerate embryos (70). Similarly, daily administration of GH increases the ovulation rate in gilts, although a long-term regimen (more than 9 days) of GH administration suppresses their subsequent estrus cycle (71). However, GH-transgenic pigs exhibit a decrease in ovulation rate (72).

The corpus luteum (CL) is a dynamic endocrine gland that plays an integral role in regulating the menstrual cycle and early pregnancy (73). Given that GH is expressed in luteal cells of multiple mammals, GH regulates luteal function by exerting its proliferative and antiapoptotic actions on luteal cells (43, 44). In this regard, GH has been shown to stimulate the proliferation of human luteinized granulosa cells and suppress apoptosis in pig corpus lutea (26, 46). Indeed, Ghr knockout mice have fewer apoptotic antral follicles (69). The supportive effect of GH on luteal function can partially reflect GH-induced progesterone production as progesterone is also an antiapoptotic factor (74, 75). Moreover, in the early stage of bovine corpus luteum, *in vitro* administration of GH with other luteal peptides increases the secretion of prostaglandin F2α, which further stimulates progesterone production and maintains the function of the corpus luteum (76).

Compared to the emphasis on potentially improved oocyte quality and quantity, little attention has been given to the effect of GH on the endometrium, despite the well-reported expression of GH and GHR in this target site (32). *In vitro* studies showed that the addition of GH to the cultured endometrial and decidual cells increased the cell proliferation rate (77). Similarly, a previous study using an *in vitro* invasion model showed that extravillous cytotrophoblasts isolated from first-trimester chorionic villi can express GHR and that GH (GH-V) stimulates extravillous cytotrophoblast cell invasiveness, indicating a uterine role of GH in the regulation of trophoblast invasion (77).

### Possible implications of IGF-1

Increasing evidence suggests that GH acts in synergy with IGF-1 to promote the differentiation of granulosa and theca cells into luteal cells (42). In particular, pituitary GH interacts with GHR and triggers the recruitment and phosphorylation of JAK2 at the cytoplasmic domain of GHR (37). As a result, this GHR/JAK2 complex subsequently phosphorylates STAT5b and upregulates IGF-1 expression in liver cells (37, 78). Similarly, this GH/GHR-induced intracellular signaling is responsible for the production of IGF-1 in rat granulosa cells (79). Although the downstream signaling cascades of FSH and LH are diverse, recent studies have demonstrated coordinated crosstalk between the FSH/LH and GH/IGF-1 signaling pathways (42).

GH is more frequently applied as an adjuvant reagent during fertility treatment, and the results from studies using animal and human models demonstrate that GH and IGF-1 are important intraovarian regulators of steroidogenesis, cell proliferation, and follicular development (42). Indeed, a prospective comparative pilot study showed that the follicular levels of GH and IGF-1 are higher in the corresponding oocytes with a normal morphology (80), indicating a correlation of follicular fluid GH/IGF-1 levels with oocyte quality. In dairy cows, intraovarian IGF-1 injection increases the cell number of the inner cell mass (ICM) during the morula to blastocyst transition of the embryos, indicating a regulatory role of IGF-1 in embryo development (81). The effect of IGF-1 on oocyte maturation has been demonstrated using a canine model showing that IGF-1 can promote oocyte maturation during *in vitro* maturation (IVM), most likely *via* the IGF-1R-mediated Ras/MAPK pathway in cumulus cells (82). Importantly, a clinical study using human immature oocytes showed that supplementation with IGF-1 (along with epidermal growth factor and brain-derived neurotrophic factor) in the cultured medium improves the maturation rate and quality of oocytes, leading to better outcomes of embryo development and blastocyst formation (83).

## Growth hormone and aging

GH plays a key role in regulating postnatal growth, metabolism, and body composition; however, deficiency in GH-related signaling results in delayed aging and extended longevity in mice (4). The evidence came from a report showing that Ames dwarf mice (*Pit1* gene mutation) have an increase in lifespan (50%) compared with their wild-type siblings, indicating that the extension of longevity is caused by GH deficiency (84). Another causative role of GH deficiency in the extension of longevity of the related mutant animals was supported by the fact that mice with isolated GH deficiency or those with GHR deletion (GH-resistant) also live much longer (85, 86). Moreover, there are delays in cognitive decline and in the onset of age-related diseases (cardiovascular diseases, diabetes, and cancers) in GH-deficient and GH-resistant animals (4). The homologous growth factor IGF-1 also contributes to the regulation of aging and lifespan, and this effect is evolutionarily conserved from worms to mammals (87). The decreased body size and reduced fertility observed in GH-deficient or GHR-depleted animals can be considered compensation for their remarkably extended longevity. Despite a large number of studies on the effects of diminished GH-related signaling on lifespan, the underlying mechanisms are not fully understood. However, the possible mechanisms include reduced inflammation, enhanced stress resistance and xenobiotic metabolism, various metabolic adjustments, and improved insulin signaling cascades (4). In humans, pathological excess of GH has been shown to reduce life expectancy, and GH resistance or deficiency provides protection from major age-related diseases (including cardiovascular diseases and cancers) in men (88–90). Nonetheless, no evidence has yet been provided to reveal increased longevity in humans with GH deficiency or GH resistance.

## Growth hormone in animal reproductive science

An animal model mimicking Laron syndrome (hereditary dwarfism resulting from defects in the *GHR* gene in humans) was developed by disrupting the mouse Ghr/binding protein (*Bp*) gene (86). GHR/BP knockout mice exhibit the absence of GHR and GH binding protein with a phenotype of severe postnatal growth retardation, dwarfism, decreased serum levels of IGF-1, and elevated serum levels of GH (86). Interestingly, previous studies using experimental designs to block or impair the action of GH on its receptor (GH-resistant or GHR- knockout mice) resulted in a similar phenotype regarding fertility (86, 91). Various fertility parameters are similarly reduced in these transgenic mice, including a delay in pubertal development, a delay in the exhaustion of the follicular pool, and a decrease in litter size (91). Further studies indicated that the decrease in litter size is due to a reduction in ovulation rate rather than detrimental effects on embryo implantation and placentation (92). For instance, ovaries obtained from these transgenic mice showed a predominant increase in primordial and primary follicles, while an absence (or limited presence) of advanced follicle stages (antral and preovulatory) was observed (86, 91, 93). Intriguingly, this subfertility phenotype can be reversed by the administration of IGF-1, indicating that IGF-1 is most likely the immediate downstream mediator responsible for the regulation of follicular function (53).

Targeted depletion of *Igf-1* in mice leads to a phenotype of dwarfism and infertility (94). These mutant mice exhibit failure in ovulation (either spontaneous or gonadotropin-induced ovulation), an increase in primordial and primary follicles and an absence of antral follicles, indicating an essential role for IGF-1 in regulating the activation of primordial follicles and the progression of early stages of follicles into gonadotropin-dependent growing follicles (94). Additionally, an *in vitro* study showed that the growth rate of mouse secondary follicles correlates with the expression levels of *Igf-1* mRNA (95).

The other approach to investigate the functional role of GH in follicular development is based on the modification of GH secretion by the pituitary gland (96). Pituitary secretion of GH is predominantly abolished in mice with targeted depletion of GH (Ames dwarf mice), leading to an increased number of primordial follicles and a dramatic reduction in antral follicles (96). However, administration of GH can reverse this situation by decreasing the number of primordial follicles and increasing the antral follicular count (96). These results support the notion that GH may enhance the process of primordial follicle activation and that ovarian follicles remain in the primordial stage in the absence of GH (92). GH also promotes the ovulation rate as GH- transgene expression or administration of GH increases the ovulation rate in mice (68). Similarly, in agricultural settings, overexpression of GH or administration of GH during ovulation (or insemination) improves ovulation and pregnancy rates in sheep, gilts, ewes, and cattle (5, 6, 70, 97).

## Growth hormone and human oocyte quality

The development of an optimal embryo and thus a successful pregnancy is principally dictated by a “good-quality” oocyte (98). Approximately 10 to 60% of the retrieved oocytes obtained from women undergoing IVF present one or more morphological abnormalities, including irregular shape, diffuse cytoplasmic vacuoles, granularity, refractile bodies, large perivitelline space, perivitelline debris, and a large or fragmented first polar body (98, 99). Although still not well characterized, such morphological abnormalities could be caused by either intrinsic factors (advanced maternal age and genetic defects) or extrinsic factors (nutrition, ovulation induction reagents, and culture conditions) (98). Given the beneficial effects (direct and IGF-1-mediated indirect) of GH on follicular development, steroidogenesis, and oocyte maturation (100, 101), research using clinical samples to evaluate the correlation of GH and oocyte quality has been a focus of great interest. Indeed, serum levels of GH and IGF-1 are positively correlated with follicular morphology, oocyte maturity, and steroid concentrations (102, 103).

Follicular fluid provides a perfect microenvironment to support the developmental competence of the oocyte and represents an optimal source for evaluating oocyte quality (104). A previous study using follicular fluid samples obtained from IVF patients showed a positive correlation between follicular GH and oocytes, giving rise to transferred embryos (105). Another study also found a positive correlation between follicular IGF-1 and the number of matured oocytes (102). With regard to oocyte quality, a recent prospective pilot study found that the follicular fluid levels of GH and IGF-1 were higher in the normal oocyte cohort than in the abnormal oocyte cohort (more than 50% of oocytes presented at least one morphological abnormality), indicating a positive correlation between the follicular fluid levels of GH and IGF-1 and oocyte quality (80). Additionally, this study also demonstrated that the fertilization rate was higher in the normal oocyte cohort than in the abnormal oocyte cohort (80). In terms of embryo quality, studies comparing follicular fluid hormones showed that higher GH concentrations in follicular fluid were positively associated with various parameters of embryo quality, including cleavage rate, good morphology, and implantation potential (105, 106). However, other studies did not show such associations, finding no significant differences in follicular IGF-1 levels obtained from follicles of different grades of oocyte maturation in humans (107, 108). The notion that higher follicular levels of GH and IGF-1 are associated with better developmental competence of oocytes has been supported by studies using GH or IGF-1 as adjuvant reagents to improve IVF outcomes. A previous randomized control study (RCT) using a GnRH agonist long protocol demonstrated that GH adjuvant therapy improved the number of retrieved oocytes, mature (MII) oocytes, fertilized oocytes, and transferred and cryopreserved embryos (109), indicating the beneficial effect of GH on oocyte quality and corresponding embryo formation. Additionally, adjuvant treatment using GH has been shown to enhance the ovarian response to stimulation reagents, increase the number of retrieved oocytes and embryos, and improve pregnancy and live birth rates (110). Furthermore, the addition of IGF-1 to the culture medium of immature oocytes during *in vitro* maturation led to an increased number of mature oocytes (111) and a reduced number of oocytes with morphological abnormalities (83). These findings highlight the beneficial effects of GH and IGF-1 on the competent development of oocytes.

## Growth hormone and human uterine receptivity

Compared to the improvement of ovarian/follicular functions, less emphasis was paid to the role of GH in the implantation site. Emerging evidence provided by recent studies indicates that GH and its functional receptors are also expressed in the endometrium, and this locally produced hormone plays a critical role in regulating endometrial receptivity and embryo implantation. During the implantation window, the endometrial tissue undergoes structural and functional changes through an integrated interaction between various endometrial cell types modulated by many growth factors and steroid hormones (112). In clinical practice, several techniques, including ultrasonography (thickness and uterine perfusion), histology, and molecular biomarkers, have been used to evaluate endometrial receptivity and predict implantation potential in infertile women undergoing embryo transfer (112, 113).

The mechanisms underlying the effects of GH on endometrial thickness and uterine perfusion remain unclear. Animal models and *in vitro* cell cultures have been used to address this question, from basic science to the development and assessment of GH in clinical application (114). Targeted depletion of *Ghr* in mice leads to a detrimental impact on reproduction during early pregnancy because of fewer uterine implantation sites (115). After GH administration, increased concentrations of cytosolic estrogen receptors have been detected in the uteri of several species, including rabbits, guinea pigs and cows, indicating a potential estrogen-mediated function (116–119). For instance, injection of GH during an ovulation induction protocol associated with timed artificial insemination increased the pregnancy rate in lactating cows (119). The GH-induced upregulation of IGF-1 in the uterus may mediate the beneficial effect of GH on endometrial thickness (115). Similarly, the addition of porcine GH increased the mRNA levels of IGF-1, IGF-2, and insulin-like growth factor binding protein 2 (IGFBP2) in the pig uterus, suggesting the functional role of the GH/IGF axis in the uterine endometrium (27, 115). Further *in vitro* experiments demonstrated differential roles of IGF-1 and IGF-2 in endometrial stromal cells, as IGF-1 mediates estrogen-induced mitogenic effects, while IGF-2 promotes endometrial differentiation (27, 115, 120). Studies using mouse models have identified several molecular biomarkers of endometrial receptivity in response to GH (114). Administration of GH in mice induced the upregulation of a variety of molecules in the endometrium, including matrix metalloproteinase 9 (Mmp-9), leukemia inhibitory factors (Lif) and integrin alpha v beta 3 (Itgavb3), which are critical regulators of embryo implantation (114). An animal study using a rat model also showed that supplementation with GH increased the expression of Itgavb3 and osteopontin, which further improved endometrial receptivity (see reviews (114)). *In vitro* studies using cell-cultured endometrial and decidual cells have demonstrated that the addition of GH into the culture medium enhanced cell proliferation (121). Similarly, overexpression of GH in endometrial carcinoma cell lines (RL95-2 and AN3 cells) led to an increase in cell number because of the enhancement of cell proliferation and suppression of apoptotic cell death *via* the upregulation of IGF-1, VEGF, and Itgb3 (77, 122). However, pretreatment with the Janus kinase (JAK) 2 inhibitor AG490 completely abolished the GH-induced upregulation of IGF-1, VEGF, and Itgb3, indicating that the JAK2 signaling pathway mediates GH action in these cells (122).

During the periovulatory phase (on the day of human chorionic gonadotropin (hCG) administration or oocyte retrieval), women with an increased endometrial thickness and uterine perfusion have been reported higher pregnancy rates following IVF (123–126). The results obtained from clinical studies indicate that supplementation with GH may enhance endometrial receptivity and improve various pregnancy outcomes in women undergoing IVF/embryo transfer (ET) (113, 127–129). Using ultrasound as a tool, changes in endometrial thickness and uterine perfusion can be evaluated among women undergoing IVF/ET (127, 129, 130). For instance, two infertile cases with panhypopituitarism have been reported to illustrate the functional role for GH in the promotion of endometrial receptivity (130, 131). Specifically, the administration of GH followed by gonadotropin treatment increased the endometrial thickness in an infertile woman with panhypopituitarism, leading to a successful pregnancy (131). However, there are controversial results showing the clinical evidence of GH on endometrial receptivity to improve pregnancy outcomes in women undergoing IVF (see reviews (114)). Despite no consistent results showing significant increases in implantation rate and pregnancy rate, there seems to be beneficial effects of GH on the number of retrieved oocytes and endometrial thickness in poor responders (114, 132, 133). A retrospective study including 1114 normal responders revealed that adjuvant GH treatment increased endometrial thickness in older patients (more than 35 years old) and improved the implantation rate and pregnancy rate among all ages (134). Similarly, the beneficial effects of GH on endometrial thickness, implantation rate and pregnancy rate have been shown in infertile women who are overweight or obese (114, 134). Studies using animal models or *in vitro* cell cultures have detected changes in several biological markers related to endometrial receptivity after GH treatment (135, 136).

## Therapeutic use of growth hormone in human female infertility

**Figure 1:**
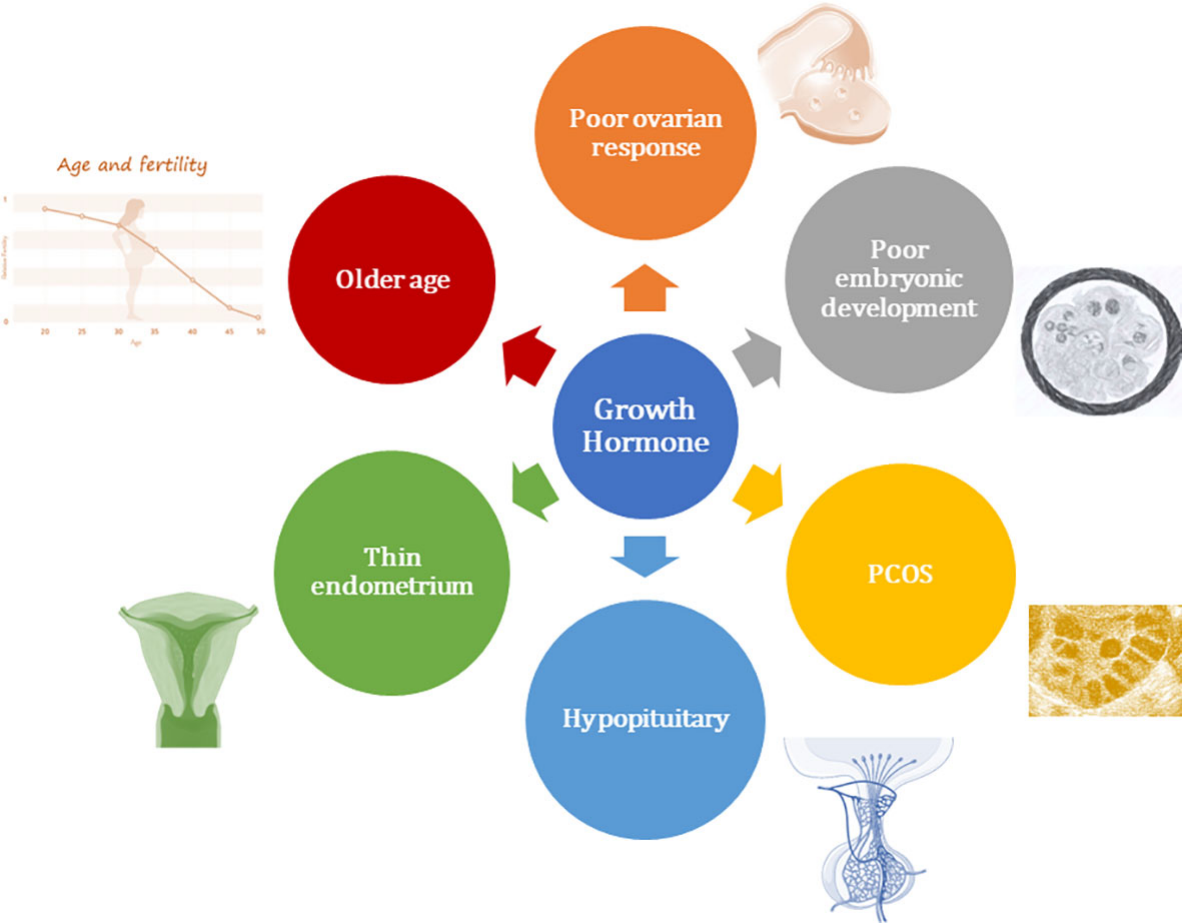
Overview of clinical application of growth hormone in women undergoing assisted reproductive technology.

Although GH has long been applied for the treatment of female infertility during ovulation induction (137), it is still controversial to address the beneficial effects of GH as an adjuvant in IVF/ET treatment (138). The debate has occurred because of the issues inherent to the relatively underpowered studies that included infertile women with poor prognosis in terms of pregnancy potential. These women have experienced several unsuccessful IVF treatment cycles due to either a poor response to ovulation induction or poor oocyte quality. The other issues raised in evaluating the role of GH in the treatment of female infertility are as follows: the optimal dosage to use; when GH treatment should be commenced; the duration of administration; and which subgroup of patients should be recruited (138). Furthermore, each study has different inclusion criteria for the subjects studied, which makes it difficult to compare studies performed in similar subsets of women undergoing fertility treatment, especially in women with poor response (139). At present, GH supplementation has been employed during assisted reproductive technology (ART) treatment in infertile women with normal response, women with poor response, women with poor embryonic development, women with oocyte donation, women with older age, women with polycystic ovarian syndrome, and even women with a thin endometrium resistant to any therapy (**Figure 1**) (8, 9, 110, 122, 137, 138, 140–143).

### Results obtained in women with poor ovarian response

Clinical studies have shown that the follicular IGF-1 concentrations of the retrieved oocytes are positively associated with the number of developing follicles and are negatively associated with the amount and duration of ovarian stimulation (144). Additionally, the follicular GH concentrations are higher in the retrieved oocytes, resulting in successful fertilization, embryo development and implantation, and clinical pregnancy (105, 106). Given its functional role in regulating follicular development and recruitment, GH has been used as an adjuvant treatment during ovulation induction for ART for more than three decades (145). For women with GH deficiency-associated infertility, GH supplementation has been used to manage their ovulation disorders (146, 147). Initially, the first two randomized controlled trials (RCTs) regarding the application of GH as an adjunct for women undergoing IVF treatment were performed three decades ago (140, 141). However, the results of these studies did not identify any better clinical outcomes associated with GH supplementation (140, 141). Therefore, subsequent studies have focused more on the addition of GH restricted to patients undergoing IVF treatment who respond poorly to ovulation induction (**Table 1**) (109, 110, 138, 148–150). Although experimental research has demonstrated apparent benefits of GH in regulating follicular functions, there is a lack of clarity regarding the potential benefit of GH supplementation in some of the clinical parameters during ART treatment because of substantial differences in the inclusion criteria among clinical studies. A previous RCT including 16 women with amenorrhea and anovulatory infertility showed that patients receiving GH treatment (24 IU on alternate days with a total dose of 144 IU) had significant decreases in the dosage of human menopausal gonadotrophin (hMG), duration of treatment, and the daily effective dosage of gonadotropins (137). Additionally, this study also showed that the serum levels of IGF-1 rose during treatment with GH but not with the control, indicating that GH may augment the response to gonadotropin stimulation in human ovaries (137). Several meta-analyses have been used to assess the value of GH supplementation in women with poor response during IVF treatment. A meta-analysis performed by Kolibianakis et al. showed that GH supplementation increased the clinical pregnancy rate and the live birth rate in women with poor response (148). However, the total number of included cases was too small (6 RCTs, including 169 patients) (148). Kyrou et al. conducted a meta-analysis including 22 RCTs and evaluated various medical interventions for IVF/ET outcomes in poor responders (149). Among 15 interventions, GH addition to ovarian stimulation and embryo transfer on Day 2 (compared with Day 3) were the only two interventions that increased the probability of pregnancy (149). Similarly, a meta-analysis (11 RCTs) by Li et al. also showed that GH addition significantly improved pregnancy outcomes by increasing the clinical pregnancy rate and live birth rate as well as the number of collected oocytes and MII oocytes (150). Another meta-analysis conducted by Yu et al. showed that GH addition increased the serum levels of estradiol (on the day of hCG administration), number of MII oocytes, number of 2PN embryos, and number of transferred embryos (151). Using a network meta-analysis, our previous study evaluated the role of different adjuvant treatment strategies on IVF treatment outcomes in poor responders (152). Our results showed that adjuvant treatment with GH had the highest number of oocytes retrieved, the highest number of embryos transferred, and the highest estradiol level on the hCG day, although there was no significant increase in the clinical pregnancy rate found compared to the control group (152). A Cochrane review performed in 2003 concluded that GH addition during IVF treatment has no certain effect on the number of retrieved oocytes and live birth rates in normal responders (153). However, GH slightly increased the number of retrieved oocytes and clinical pregnancy rates in women with poor response (153). Nonetheless, there is an uncertain effect of GH addition on live birth rates in poor responders because the dosage and regimen of GH therapy used in trial studies were variable (153). Although the results obtained from the meta-analysis suggested that GH addition can improve early clinical outcomes, the beneficial effect of GH on live birth is still widely debated. A large-scale retrospective study including 3,080 poor responders receiving IVF treatment demonstrated that GH treatment may not improve the live birth rate in expected poor responders (154). These inconsistent results obtained from different studies need to be interpreted with caution. Some of the included trials were not RCTs and may have significant bias and imprecision. Additionally, the dosage and regimen of GH supplementation used in every study were quite variable. Future comprehensive research including more high-quality RCTs is required to determine the role of GH as an adjuvant reagent for poor responders during their IVF treatment.

**Table 1:** Studies of randomized control trial using growth hormone in a population of poor ovarian responders.

Trial	Study design	Age/BMI	Poor response type	GH cases	GH	Dose	Duration	Outcome
				Control cases	Protocol			
Owen 1991 (1)	Single-center RCT	<38	PCOS patient <6 oocytes previous cycle or <4 embryos developed	13	HGH	24 IU	Alternate day from D1 stimulation (maximum 2 weeks)	Duration of hMG *Total dosage of hMG ↓ *No. of follicles> =14 mm ↑ No. of MII oocytes *Fertilization rate ↑ No. of cleavage embryos (p< 0.02) No. of oocytes retrieved (p< 0.03) Pregnancy rate
				12	GnRH agonist			
Younis 1992 (2)	A prospective randomized placebo-controlled double-blind study	<38	N	20	HGH	12 IU	Days 1, 3, 5, 7	Follicular phase duration hMG ampules used serum E2 number of follicles (~14 mm) on day of hCG number of oocytes and embryos achieved Embryo morphology rate of cleavage Clinical pregnancy rate (PR) per embryo transfer Implantation rate
				22	Long protocol			
Tapanainen 1992 (3)	A prospective randomized placebo-controlled study	27-37	ovarian hyperstimulation	19	HGH	24 IU	Alternate day from Day 4 stimulation until the last HMG	*E2 and P level on the day of hCG injection ↓ LH, FSH, prolactin, testosterone (T), and sex hormone-binding globulin levels *T levels n the follicular fluid (FF) ↑ E2, P, IGF-I levels in the FF No. of hMG ampules No. of follicles and oocytes recovered
				19	Flare			
				15	Agonist			
Bergh 1994 (4)	A multicenter RCT	25-38/ <28 kg/m2	normal ovulatory patient: <5 oocytes retrieved previous IVF cycles x2	I: placebo/placebo: 10; II: placebo/GH: 10; III: GH/GH: 10; IV: GH/placebo: 10;	HGH	0.1 IU/kg	Pretreatment for 7 days followed by with/without stimulation	Number of oocytes Duration of hMG Total dosage of hMG E2 levels *serum and FF levels of IGF-I. Endometrial thickness No. of embryos Pregnancy rate
					Long protocol			
Dor 1995 (5)	A single-center RCT		Response*2 estradiol < 500 pg/ml, <3 oocytes retrieve	7	HGH	18 IU	On Days 2, 4, 6, 8	Total dosage of hMG E2 level No. of oocytes Fertilization rate No. of embryos transferred
				7	Flare			
Suikkari 1996 (6)	A single-center RCT	<40/ <27 kg/m2	Poor response *2	group 1: 10 group 2: 6	HGH	3 groups 4 IU 12 IU, placebo	Daily from Day 3	Cancelation rate total dosage of FSH E2 levels No. of oocyte Fertilization rate Implantation rate Pregnancy rate
				group 3: 6	Flare			
Kucuk 2008 (7)	A single-center RCT		Response	31	HGH	12 IU	Daily from Day 21 of the previous cycle	Duration of stimulation Total dosage of FSH Cost of COS E2 levels No. of MII oocytes No. of transferred embryos Implantation rate Pregnancy rate
				30	Agonist			
Eftekhar 2012 (8)	A single-center RCT	≤ 30 kg/m2	Response	40	HGH	4 IU	Daily injection from day 21 of the previous cycle until the day of hCG injection	Duration of stimulation Total dosage of HMG Endometrial thickness E2 level Cancellation rate No. of MII oocytes No. of oocyte retrieved No. of embryos No. of embryos transferred Fertilization rate Implantation rate Biochemical pregnancy rate Clinical pregnancy rate Miscarriage rate
				42	Antagonist			
Bayoumi 2015 (9)	A single-center RCT	N/A	Bologna	84	HGH	2.5 mg	Daily from Day 6 of hMG stimulation until ovulation	Total dosage of hMG Duration of stimulation Endometrial thickness E2 level *No. of oocytes *No. of MII oocytes *Fertilization rate No. of embryos transferred Implantation rate Chemical pregnancy rate Clinical pregnancy Cycle cancellation rate
				88	Microflare			
Bassiouny et al. 2016 (10)	A single-center RCT	N/A	Bologna	68	HGH	2.5 mg	Daily from Day 6 of hMG stimulation until the day of hCG trigger	*E2 level *No. of MII oocytes *No. of oocytes *No. of fertilized oocytes *No. of transferred embyos clinical pregnancy rates per cycle (-), early miscarriage rate, ongoing pregnancy live birth rates per cycle (-)
				73	antagonist			
Choe 2017 (11)	A single-center RCT	≥ 40/ < 30 kg/m2	Bologna	62	Sustained-release GH	20 mg	A total of 3 doses from the previous cycle mid luteal, late luteal and Day 2	*No. of oocytes E2 levels *No. of MII oocytes No. of good-quality embryos Fertilization rate Implantation rate Clinical pregnancy rate Ongoing pregnancy rate Miscarriage rate
				65	Antagonist			
Dakhly 2018 (12)	A single-center RCT	≥ 40	Bologna	120	HGH	7.5 IU	Daily from Day 21 of the previous cycle	Duration of stimulation dosage of gonadotropins E2 levels Endometrial thickness *No. of oocytes *No. of MII oocyte *Fertilization rate Implantation rate *No. of transferred embryos *No. of frozen embryos Cancelled cycles Chemical pregnancy rate Clinical pregnancy rate Miscarriage rate Ongoing pregnancy rate Live birth rate (fresh or cumulative)
				120	Long protocol			
Moham- mad 2019 (13)	A randomized controlled study	25-38	Response	78	HGH	4 IU	Day 2 until 1 day before egg collection	*E2 levels *No. of oocyte retrieved *No. of MII oocytes Fertilization rate, Implantation rate, Pregnancy rate
				78	Antagonist			
Norman 2019 (14)	A multicenter RCT	< 41 /<33 kg/m2	Response	65->62	HGH	12 IU	Day 1 until 1 day before egg collection	Total dosage of rFSH Duration of stimulation No. of oocyte retrieved Fertilization rate No. of embryos transferred No. of embryos cryopreserved Quality of embryos obtained Miscarriage rate Live birth rate
				65->51	Antagonist			
Safdarian 2019 (15)	Single-center RCT		Bologna	group A: 34 group B: 32	HGH	groups: A. 7.5 IU from day 8 B. 0.3 IU from day 3 previous cycle C. saline form day 8	3 groups: A. 7.5 IU from Day 8 for 5 days B. 0.3 IU from Day 3 previous cycle for 20 days C. saline from Day 8 for 5 days	Total dosage of rFSH Duration of stimulation Endometrial thickness *No. of oocyte retrieved *No. of MII oocytes *No. of embryos transferred *Fertilization rate *clinical pregnancy rate *Live birth rate (B)
				group C: 26	Antagonist			
Gong 2020 (16)	A single-center RCT	33-43	Bologna	group A: 57	HGH	4 IU/d	On day 2 of the previous menstrual cycle before IVF until the trigger day	POR-GH V.S. POR *Endometrial thickness on trigger day *No. cleaved embryos *No. of higher-quality embryos *Embryo formation, *Quality embryo formation, *Implantation rate *Clinical pregnancy
				group B (POR):60 group C (non-POR): 58	Antagonist			
Mohammad, 2021 (17)	A single-center RCT	25-38	Response*2	78 78	HGH Antagonist	4 IU/day	Day 2 until 1 day before egg collection	*E2 levels *No. of oocyte retrieved *No. of MII oocytes Fertilization rate Implantation rate pregnancy rate

* Statistical significant after using growth hormone in a population of poor ovary responders (P-value <0.05).

### Results obtained in women with poor oocyte quality or poor embryonic development

Poor oocyte quality and subsequent poor embryonic development are the major causes of treatment failure after IVF/ET, especially in women with a normal response, as there is a close association between oocyte/embryo quality and pregnancy rate (155, 156). Embryo development at the early stage is highly dependent on oocyte-derived substances, including mRNAs, proteins, and mitochondria, which are essential for successful implantation (157, 158). Experimental results obtained from animal models and clinical samples have shown that GH can improve mitochondrial function, cytoplasmic maturation, and nuclear maturation of oocytes in mice, women with poor response, and women with advanced ages; thus, GH addition increases the number of matured oocytes retrieved and subsequent embryo quality (41, 148, 159). An RCT conducted by Li et al. recruited 158 patients with at least one previous IVF cycle failure due to a lack of top-quality embryos (160). Using a daily dose of 3 IU human GH (initial day of downregulation for the long protocol or stimulation day for the antagonist protocol until the hCG day), the results showed that GH supplementation increased the number of retrieved oocytes and cleaved embryos, leading to a higher implantation rate, clinical pregnancy rate, and live birth rate (160). Additionally, the cumulus granulosa cells obtained from the GH addition group had higher mitochondrial DNA copy numbers than those obtained from the control group, indicating that GH may affect the oocyte quality and developmental competence (160). Similarly, a previous RCT conducted by Dakhly et al. demonstrated that GH adjuvant therapy improved the number of retrieved oocytes, mature (MII) oocytes, fertilized oocytes, and embryos reaching the transfer stage (109), indicating a beneficial effect of GH on oocyte quality and corresponding embryo formation. Indeed, a Cochrane review by Duffy et al. summarized that adjuvant treatment using GH enhanced ovarian response to ovulation stimulation, increased the number of retrieved oocytes and embryos, and thus improved pregnancy rate and live birth rate (110).

### Results obtained in women receiving donated oocytes

Successful implantation is a complex process that principally depends on the synchronization of good-quality embryos with the receptive endometrium (161). Inadequate uterine function accounts for approximately two-thirds of implantation failures, and poor embryo quality accounts for one-third of these failures (162). In addition to the ovary, the uterus is the other target site of GH action (7). Animal studies have demonstrated that GH not only enhances embryonic development but also increases the post-transferred pregnancy rate in embryo-recipient cows (163). The first RCT study (including 240 patients) in humans showing the beneficial effect of GH on the endometrium, indicating that GH addition associated with hormone replacement therapy can improve clinical outcomes in patients with frozen embryo transfer (129). Specifically, the endometrial thickness, uterine perfusion, and cytokine expression profile related to endometrial receptivity were greater in the GH group than in the control group (129).

Oocyte donation has expanded the scope of ART for women with poor oocyte quantity and quality. Despite the relatively high success rate of oocyte donation programs in recipients, some patients still encounter implantation failure repeatedly (164). An RCT (including 70 women with repeated implantation failure with donated oocytes) was designed to evaluate the GH effect on uterine receptivity and found that administration of GH increased endometrial thickness, leading to a higher pregnancy rate and live birth rate (128). Similarly, a recent meta-analysis including 25 RCTs with 2,424 women evaluated whether GH administration can improve endometrial function and reproductive outcomes during IVF treatment (10). Administration of GH significantly increased endometrial thickness, which accounted for an enhanced clinical pregnancy rate and live birth rate (10). Additionally, there existed a dose- and time-dependent effect of GH on IVF outcomes, indicating that GH might improve the fertility outcome in the recipients through its effect on the endometrium rather than on oocytes or embryos (10). Moreover, further GH regimen analysis showed differential effects on the endometrium and oocytes, as < 5 IU/day GH administration from the follicular phase of the previous cycle until the hCG day resulted in a thicker endometrium, while 5–10 IU/day or administration from the luteal phase of the previous cycle until the hCG day led to better quality of oocytes and embryos (10). Data obtained from four RCTs on women with a thin endometrium suggested that improving endometrial function for these patients is essential for their ART outcomes (10). In this regard, GH administration might improve endometrial function by affecting several parameters, including endometrial thickness, endometrial morphology, and uterine perfusion (10). However, further studies of GH effects on human uterine receptivity will be required before providing any clinical recommendations in ART protocols.

### The safety and potential risks of GH administration in ART

Currently short-term use of GH in ART is not expected to have any problems or safety concerns; however, GH administration may potentially have side effects on active cancers and some metabolic diseases, such as diabetes mellitus (165, 166). Indeed, GH administration has been shown to lead to significant metabolic changes, including insulin resistance, glucose intolerance, elevated cholesterol, and disturbance of the renin-angiotensin system (166–168). However, van Bunderen et al. conducted a systematic review and found that long-term administration of GH had a beneficial effect on decreasing the risk of fracture, cardiovascular disease, and stroke without increasing the risk of malignancy (169). Nonetheless, clinical practitioners should consider the potential risks and comprehensively individualized assessment of the dosage and regimen for the application of GH during ART treatment.

## Conclusion

GH has been considered an adjuvant treatment in human ART for three decades. In addition to the anterior pituitary gland, GH and its functional receptor are also expressed in the female reproductive system, including the ovaries and uterus. Emerging evidence supported by animal studies and clinical trials in humans has suggested that GH and IGF-1 play essential roles in the regulation of multiple ovarian functions, ranging from activation of the primordial follicles, folliculogenesis, ovarian steroidogenesis, oocyte maturation, and embryo implantation. The follicular concentrations of GH and IGF-1 are positively correlated with oocyte quality and the subsequent embryo morphology and cleavage rate. Clinical trials have demonstrated that GH intervention is associated with improved IVF reproductive outcomes, including the number of mature oocytes retrieved, fertilization rate, embryo quality, implantation rate, pregnancy rate, and live birth rate, especially in patients with poor ovarian response. Additionally, women with thin endometrium and recurrent implantation failure might benefit from GH administration. However, discrepancies exist between studies because of methodological weaknesses and limited sample sizes, leading to a lack of clinical recommendations for the application of optimal GH regimens during IVF treatment. More RCTs with rigorous methodology are needed to confirm the beneficial effects of GH on ART outcomes.

## Author contributions

C-WC collected the information and designed the figure and table, and wrote the manuscript. Y-WS and Y-WH designed the figure. Y-YC, MH, H-CH, T-CY, and W-CL contributed to the critical discussion. H-MC critically and revised the manuscript and contributed to the conception of design. All authors contributed to the article and approved the submitted version.

## Conflict of interest

The authors declare that the research was conducted in the absence of any commercial or financial relationships that could be construed as a potential conflict of interest.

## Publisher’s note

All claims expressed in this article are solely those of the authors and do not necessarily represent those of their affiliated organizations, or those of the publisher, the editors and the reviewers. Any product that may be evaluated in this article, or claim that may be made by its manufacturer, is not guaranteed or endorsed by the publisher.

## References

[1] Harper ME Barrera-Saldana HA Saunders GF. Chromosomal localization of the human placental lactogen-growth hormone gene cluster to 17q22-24. Am J Hum Genet (1982) 34(2):227–34. PMC1685277 7072716

[2] de Boer JA Schoemaker J van der Veen EA. Impaired reproductive function in women treated for growth hormone deficiency during childhood. Clin Endocrinol (Oxf) (1997) 46(6):681–9. doi: 10.1046/j.1365-2265.1997.1800999.x 9274698

[3] Giampietro A Milardi D Bianchi A Fusco A Cimino V Valle D. The effect of treatment with growth hormone on fertility outcome in eugonadal women with growth hormone deficiency: report of four cases and review of the literature. Fertil Steril (2009) 91(3):930.e7–11. doi: 10.1016/j.fertnstert.2008.09.065 19046578

[4] Bartke A Sun LY Longo V. Somatotropic signaling: trade-offs between growth, reproductive development, and longevity. Physiol Rev (2013) 93(2):571–98. doi: 10.1152/physrev.00006.2012 PMC3768106 23589828

[5] Moreira F Orlandi C Risco CA Mattos R Lopes F Thatcher WW. Effects of presynchronization and bovine somatotropin on pregnancy rates to a timed artificial insemination protocol in lactating dairy cows. J Dairy Sci (2001) 84(7):1646–59. doi: 10.3168/jds.S0022-0302(01)74600-0 11467815

[6] Starbuck MJ Inskeep EK Dailey RA. Effect of a single growth hormone (rbST) treatment at breeding on conception rates and pregnancy retention in dairy and beef cattle. Anim Reprod Sci (2006) 93(3-4):349–59. doi: 10.1016/j.anireprosci.2005.08.010 16183219

[7] Hull KL Harvey S. Growth hormone and reproduction: a review of endocrine and autocrine/paracrine interactions. Int J Endocrinol (2014) 2014:234014. doi: 10.1155/2014/234014 25580121 PMC4279787

[8] Regan SLP Knight PG Yovich JL Arfuso F Dharmarajan A. Growth hormone during *in vitro* fertilization in older women modulates the density of receptors in granulosa cells, with improved pregnancy outcomes. Fertil Steril (2018) 110(7):1298–310. doi: 10.1016/j.fertnstert.2018.08.018 30503129

[9] Hazout A Junca A Menezo Y Demouzon J Cohen-Bacrie P. Effect of growth hormone on oocyte competence in patients with multiple IVF failures. Reprod BioMed Online (2009) 18(5):664–70. doi: 10.1016/S1472-6483(10)60011-9 19549445

[10] Shang Y Wu M He R Ye Y Sun X. Administration of growth hormone improves endometrial function in women undergoing in vitro fertilization: a systematic review and meta-analysis. Hum Reprod Update (2022) 28(6):838–57. doi: 10.1093/humupd/dmac028 35641113

[11] Parks JS Abdul-Latif H Kinoshita E Meacham LR Pfaffle RW Brown MR. Genetics of growth hormone gene expression. Horm Res (1993) 40(1-3):54–61. doi: 10.1159/000183768 8300051

[12] Szeto DP Ryan AK O'Connell SM Rosenfeld MG. P-OTX: a PIT-1-interacting homeodomain factor expressed during anterior pituitary gland development. Proc Natl Acad Sci U.S.A. (1996) 93(15):7706–10. doi: 10.1073/pnas.93.15.7706 PMC38811 8755540

[13] Takahashi I Takahashi T Komatsu M Sato T Takada G. An exonic mutation of the GH-1 gene causing familial isolated growth hormone deficiency type II. Clin Genet (2002) 61(3):222–5. doi: 10.1034/j.1399-0004.2002.610310.x 12000366

[14] Cohen LE Wondisford FE Radovick S. Role of pit-1 in the gene expression of growth hormone, prolactin, and thyrotropin. Endocrinol Metab Clin North Am (1996) 25(3):523–40. doi: 10.1016/S0889-8529(05)70339-X 8879985

[15] Karin M Theill L Castrillo JL McCormick A Brady H. Tissue-specific expression of the growth hormone gene and its control by growth hormone factor-1. Recent Prog Horm Res (1990) 46:43–57. doi: 10.1016/B978-0-12-571146-3.50006-7 2281188

[16] Harvey S Baudet ML. Extrapituitary growth hormone and growth? Gen Comp Endocrinol (2014) 205:55–61. doi: 10.1016/j.ygcen.2014.03.041 24746676

[17] Harvey S. Extrapituitary growth hormone. Endocrine (2010) 38(3):335–59. doi: 10.1007/s12020-010-9403-8 20972718

[18] Schwarzler P Untergasser G Hermann M Dirnhofer S Abendstein B Madersbacher S. Selective growth hormone/placental lactogen gene transcription and hormone production in pre- and postmenopausal human ovaries. J Clin Endocrinol Metab (1997) 82(10):3337–41. doi: 10.1210/jc.82.10.3337 9329365

[19] Abir R Garor R Felz C Nitke S Krissi H Fisch B. Growth hormone and its receptor in human ovaries from fetuses and adults. Fertil Steril (2008) 90(4 Suppl):1333–9. doi: 10.1016/j.fertnstert.2007.08.011 18054353

[20] Silva JR Figueiredo JR van den Hurk R. Involvement of growth hormone (GH) and insulin-like growth factor (IGF) system in ovarian folliculogenesis. Theriogenology (2009) 71(8):1193–208. doi: 10.1016/j.theriogenology.2008.12.015 19193432

[21] Slater M Cooper M Murphy CR. Human growth hormone and interleukin-6 are upregulated in endometriosis and endometrioid adenocarcinoma. Acta Histochem (2006) 108(1):13–8. doi: 10.1016/j.acthis.2006.01.004 16564564

[22] Kolle S Sinowatz F Boie G Lincoln D. Developmental changes in the expression of the growth hormone receptor messenger ribonucleic acid and protein in the bovine ovary. Biol Reprod (1998) 59(4):836–42. doi: 10.1095/biolreprod59.4.836 9746733

[23] Quesnel H. Localization of binding sites for IGF-I, insulin and GH in the sow ovary. J Endocrinol (1999) 163(2):363–72. doi: 10.1677/joe.0.1630363 10556787

[24] Lobie PE Breipohl W Aragon JG Waters MJ. Cellular localization of the growth hormone receptor/binding protein in the male and female reproductive systems. Endocrinology (1990) 126(4):2214–21. doi: 10.1210/endo-126-4-2214 2156686

[25] Yuan W Lucy MC. Messenger ribonucleic acid expression for growth hormone receptor, luteinizing hormone receptor, and steroidogenic enzymes during the estrous cycle and pregnancy in porcine and bovine corpora lutea. Domest Anim Endocrinol (1996) 13(5):431–44. doi: 10.1016/0739-7240(96)00073-2 8886596

[26] Ovesen P Ingerslev HJ Orskov H Ledet T. Effect of growth hormone on steroidogenesis, insulin-like growth factor-I (IGF-I) and IGF-binding protein-1 production and DNA synthesis in cultured human luteinized granulosa cells. J Endocrinol (1994) 140(2):313–9. doi: 10.1677/joe.0.1400313 7513345

[27] Rhoads ML Meyer JP Kolath SJ Lamberson WR Lucy MC. Growth hormone receptor, insulin-like growth factor (IGF)-1, and IGF-binding protein-2 expression in the reproductive tissues of early postpartum dairy cows. J Dairy Sci (2008) 91(5):1802–13. doi: 10.3168/jds.2007-0664 18420611

[28] Sharara FI Bhartiya D Nieman LK. Growth hormone receptor gene expression in the mouse uterus: modulation by gonadal steroids. J Soc Gynecol Investig (1994) 1(4):285–9. doi: 10.1177/107155769400100407 9419785

[29] Vroemen SF Van Marrewijk WJ De Meijer J Van den Broek AT Van der Horst DJ. Differential induction of inositol phosphate metabolism by three adipokinetic hormones. Mol Cell Endocrinol (1997) 130(1-2):131–9. doi: 10.1016/S0303-7207(97)00083-X 9220029

[30] Freese LG Rehfeldt C Fuerbass R Kuhn G Okamura CS Ender K. Exogenous somatotropin alters IGF axis in porcine endometrium and placenta. Domest Anim Endocrinol (2005) 29(3):457–75. doi: 10.1016/j.domaniend.2005.02.012 16153497

[31] Sbracia M Scarpellini F Poverini R Alo PL Rossi G Di Tondo U. Immunohistochemical localization of the growth hormone in human endometrium and decidua. Am J Reprod Immunol (2004) 51(2):112–6. doi: 10.1046/j.8755-8920.2003.00127.x 14748836

[32] Sharara FI Nieman LK. Growth hormone receptor messenger ribonucleic acid expression in leiomyoma and surrounding myometrium. Am J Obstet Gynecol (1995) 173(3 Pt 1):814–9. doi: 10.1016/0002-9378(95)90346-1 7573249

[33] Yigiter M Halici Z Odabasoglu F Keles ON Atalay F Unal B. Growth hormone reduces tissue damage in rat ovaries subjected to torsion and detorsion: biochemical and histopathologic evaluation. Eur J Obstet Gynecol Reprod Biol (2011) 157(1):94–100. doi: 10.1016/j.ejogrb.2011.02.012 21439711

[34] Chang HM Qiao J Leung PC. Oocyte-somatic cell interactions in the human ovary-novel role of bone morphogenetic proteins and growth differentiation factors. Hum Reprod Update (2016) 23(1):1–18. doi: 10.1093/humupd/dmw039 27797914 PMC5155571

[35] Chang HM Qiao J Leung PC. Neurotrophins and glial cell line-derived neurotrophic factor in the ovary: physiological and pathophysiological implications. Hum Reprod Update (2019) 25(2):224–42. doi: 10.1093/humupd/dmy047 PMC6390169 30608586

[36] van den Eijnden MJ Strous GJ. Autocrine growth hormone: effects on growth hormone receptor trafficking and signaling. Mol Endocrinol (2007) 21(11):2832–46. doi: 10.1210/me.2007-0092 17666586

[37] Rotwein P. Mapping the growth hormone–Stat5b–IGF-I transcriptional circuit. Trends Endocrinol Metab (2012) 23(4):186–93. doi: 10.1016/j.tem.2012.01.001 PMC3313013 22361342

[38] Orisaka M Miyazaki Y Shirafuji A Tamamura C Tsuyoshi H Tsang BK. The role of pituitary gonadotropins and intraovarian regulators in follicle development: A mini-review. Reprod Med Biol (2021) 20(2):169–75. doi: 10.1002/rmb2.12371 PMC8022101 33850449

[39] Jia XC Kalmijn J Hsueh AJ. Growth hormone enhances follicle-stimulating hormone-induced differentiation of cultured rat granulosa cells. Endocrinology (1986) 118(4):1401–9. doi: 10.1210/endo-118-4-1401 3004913

[40] Nimrod A. The induction of ovarian LH-receptors by FSH is mediated by cyclic AMP. FEBS Lett (1981) 131(1):31–3. doi: 10.1016/0014-5793(81)80880-0 6269897

[41] Weall BM Al-Samerria S Conceicao J Yovich JL Almahbobi G. A direct action for GH in improvement of oocyte quality in poor-responder patients. Reproduction (2015) 149(2):147–54. doi: 10.1530/REP-14-0494 25376626

[42] Ipsa E Cruzat VF Kagize JN Yovich JL Keane KN. Growth hormone and insulin-like growth factor action in reproductive tissues. Front Endocrinol (Lausanne) (2019) 10:777. doi: 10.3389/fendo.2019.00777 31781044 PMC6861326

[43] Hull KL Harvey S. Growth hormone: roles in female reproduction. J Endocrinol (2001) 168(1):1–23. doi: 10.1677/joe.0.1680001 11139766

[44] Sirotkin AV. Control of reproductive processes by growth hormone: extra- and intracellular mechanisms. Vet J (2005) 170(3):307–17. doi: 10.1016/j.tvjl.2004.05.014 16266845

[45] Doldi N Bassan M Bonzi V Ferrari A. Effects of growth hormone and growth hormone-releasing hormone on steroid synthesis in cultured human luteinizing granulosa cells. Gynecol Endocrinol (1996) 10(2):101–8. doi: 10.3109/09513599609097899 8701783

[46] Gregoraszczuk EL Ptak A. In vitro effect of leptin on growth hormone (GH)- and insulin-like growth factor-I (IGF-i)-stimulated progesterone secretion and apoptosis in developing and mature corpora lutea of pig ovaries. J Reprod Dev (2005) 51(6):727–33. doi: 10.1262/jrd.17038 16177544

[47] Gregoraszczuk EL Ptak A Wojtowicz AK Gorska T Nowak KW. Estrus cycle-dependent action of leptin on basal and GH or IGF-I stimulated steroid secretion by whole porcine follicles. Endocr Regul (2004) 38(1):15–21. 15147234

[48] Nakamura E Otsuka F Inagaki K Miyoshi T Matsumoto Y Ogura K. Mutual regulation of growth hormone and bone morphogenetic protein system in steroidogenesis by rat granulosa cells. Endocrinology (2012) 153(1):469–80. doi: 10.1210/en.2011-1646 22067323

[49] Apa R Caruso A Andreani CL Miceli F Lazzarin N Mastrandrea M. Growth hormone stimulates androsterone synthesis by rat theca-interstitial cells. Mol Cell Endocrinol (1996) 118(1-2):95–101. doi: 10.1016/0303-7207(96)03769-0 8735595

[50] Singh AK Lal B. Seasonal and circadian time-dependent dual action of GH on somatic growth and ovarian development in the Asian catfish, clarias batrachus (Linn.): role of temperature. Gen Comp Endocrinol (2008) 159(1):98–106. doi: 10.1016/j.ygcen.2008.08.001 18761011

[51] Kezele P Nilsson E Skinner MK. Cell-cell interactions in primordial follicle assembly and development. Front Biosci (2002) 7:d1990–6. doi: 10.2741/kezele 12161345

[52] Bachelot A Monget P Imbert-Bollore P Coshigano K Kopchick JJ Kelly PA. Growth hormone is required for ovarian follicular growth. Endocrinology (2002) 143(10):4104–12. doi: 10.1210/en.2002-220087 12239122

[53] Slot KA Kastelijn J Bachelot A Kelly PA Binart N Teerds KJ. Reduced recruitment and survival of primordial and growing follicles in GH receptor-deficient mice. Reproduction (2006) 131(3):525–32. doi: 10.1530/rep.1.00946 16514195

[54] Semiz O Evirgen O. The effect of growth hormone on ovarian follicular response and oocyte nuclear maturation in young and aged mice. Acta Histochem (2009) 111(2):104–11. doi: 10.1016/j.acthis.2008.04.007 18674800

[55] Sa Filho MF Carvalho NA Gimenes LU Torres-Junior JR Nasser LF Tonhati H. Effect of recombinant bovine somatotropin (bST) on follicular population and on *in vitro* buffalo embryo production. Anim Reprod Sci (2009) 113(1-4):51–9. doi: 10.1016/j.anireprosci.2008.06.008 18691835

[56] Gonzalez-Anover P Encinas T Garcia-Garcia RM Veiga-Lopez A Cocero MJ McNeilly AS. Ovarian response in sheep superovulated after pretreatment with growth hormone and GnRH antagonists is weakened by failures in oocyte maturation. Zygote (2004) 12(4):301–4. doi: 10.1017/S096719940400293X 15751538

[57] Chase CC Jr. Kirby CJ Hammond AC Olson TA Lucy MC. Patterns of ovarian growth and development in cattle with a growth hormone receptor deficiency. J Anim Sci (1998) 76(1):212–9. doi: 10.2527/1998.761212x 9464901

[58] Heikinheimo O Gibbons WE. The molecular mechanisms of oocyte maturation and early embryonic development are unveiling new insights into reproductive medicine. Mol Hum Reprod (1998) 4(8):745–56. doi: 10.1093/molehr/4.8.745 9733431

[59] Eppig JJ. Coordination of nuclear and cytoplasmic oocyte maturation in eutherian mammals. Reprod Fertil Dev (1996) 8(4):485–9. doi: 10.1071/RD9960485 8870074

[60] Pereira GR Lorenzo PL Carneiro GF Ball BA Goncalves PB Pegoraro LM. The effect of growth hormone (GH) and insulin-like growth factor-I (IGF-I) on *in vitro* maturation of equine oocytes. Zygote (2012) 20(4):353–60. doi: 10.1017/S0967199411000335 21794202

[61] Chigioni S Secchi C Borromeo V Modina S Beretta MS Luvoni GC. Effects of growth hormone on oocyte *in vitro* maturation and its localization in the canine cumulus-oocyte complexes. Vet Res Commun (2008) 32 Suppl 1:S131–4. doi: 10.1007/s11259-008-9098-y 18685999

[62] Mtango NR Varisanga MD Dong YJ Rajamahendran R Suzuki T. Growth factors and growth hormone enhance *in vitro* embryo production and post-thaw survival of vitrified bovine blastocysts. Theriogenology (2003) 59(5-6):1393–402. doi: 10.1016/S0093-691X(02)01163-9 12527085

[63] Shirazi A Shams-Esfandabadi N Ahmadi E Heidari B. Effects of growth hormone on nuclear maturation of ovine oocytes and subsequent embryo development. Reprod Domest Anim (2010) 45(3):530–6. doi: 10.1111/j.1439-0531.2008.01290.x 19032427

[64] Songsasen N Yu I Leibo SP. Nuclear maturation of canine oocytes cultured in protein-free media. Mol Reprod Dev (2002) 62(3):407–15. doi: 10.1002/mrd.10130 12112606

[65] Kolle S Stojkovic M Boie G Wolf E Sinowatz F. Growth hormone-related effects on apoptosis, mitosis, and expression of connexin 43 in bovine *in vitro* maturation cumulus-oocyte complexes. Biol Reprod (2003) 68(5):1584–9. doi: 10.1095/biolreprod.102.010264 12606495

[66] Pereira GR Lorenzo PL Carneiro GF Ball BA Bilodeau-Goeseels S Kastelic J. The involvement of growth hormone in equine oocyte maturation, receptor localization and steroid production by cumulus-oocyte complexes *in vitro* . Res Vet Sci (2013) 95(2):667–74. doi: 10.1016/j.rvsc.2013.06.024 23891385

[67] de Prada JK VandeVoort CA. Growth hormone and *in vitro* maturation of rhesus macaque oocytes and subsequent embryo development. J Assist Reprod Genet (2008) 25(4):145–58. doi: 10.1007/s10815-008-9208-3 PMC2582076 18278582

[68] Cecim M Kerr J Bartke A. Effects of bovine growth hormone (bGH) transgene expression or bGH treatment on reproductive functions in female mice. Biol Reprod (1995) 52(5):1144–8. doi: 10.1095/biolreprod52.5.1144 7626714

[69] Zaczek D Hammond J Suen L Wandji S Service D Bartke A. Impact of growth hormone resistance on female reproductive function: new insights from growth hormone receptor knockout mice. Biol Reprod (2002) 67(4):1115–24. doi: 10.1095/biolreprod67.4.1115 12297526

[70] Folch J Ramon JP Cocero MJ Alabart JL Beckers JF. Exogenous growth hormone improves the number of transferable embryos in superovulated ewes. Theriogenology (2001) 55(9):1777–85. doi: 10.1016/S0093-691X(01)00520-9 11414483

[71] Andres CJ Green ML Clapper JA Cline TR Diekman MA. Influence of daily injections of porcine somatotropin on growth, puberty, and reproduction in gilts. J Anim Sci (1991) 69(9):3754–61. doi: 10.2527/1991.6993754x 1938656

[72] Pursel VG Bolt DJ Miller KF Pinkert CA Hammer RE Palmiter RD. Expression and performance in transgenic pigs. J Reprod Fertil Suppl (1990) 40:235–45. 2192041

[73] Baerwald AR Adams GP Pierson RA. Form and function of the corpus luteum during the human menstrual cycle. Ultrasound Obstet Gynecol (2005) 25(5):498–507. doi: 10.1002/uog.1891 15846762 PMC2882116

[74] Berisha B Schams D. Ovarian function in ruminants. Domest Anim Endocrinol (2005) 29(2):305–17. doi: 10.1016/j.domaniend.2005.02.035 15998502

[75] Schams D Berisha B. Regulation of corpus luteum function in cattle–an overview. Reprod Domest Anim (2004) 39(4):241–51. doi: 10.1111/j.1439-0531.2004.00509.x 15225277

[76] Kobayashi S Miyamoto A Berisha B Schams D. Growth hormone, but not luteinizing hormone, acts with luteal peptides on prostaglandin F2alpha and progesterone secretion by bovine corpora lutea *in vitro* . Prostaglandins Other Lipid Mediat (2001) 63(3):79–92. doi: 10.1016/S0090-6980(00)00099-X 11204740

[77] Pandey V Perry JK Mohankumar KM Kong XJ Liu SM Wu ZS. Autocrine human growth hormone stimulates oncogenicity of endometrial carcinoma cells. Endocrinology (2008) 149(8):3909–19. doi: 10.1210/en.2008-0286 PMC2488240 18450952

[78] Takahashi Y. The role of growth hormone and insulin-like growth factor-I in the liver. Int J Mol Sci (2017) 18(7):1447–59. doi: 10.3390/ijms18071447 PMC5535938 28678199

[79] Dehkhoda F Lee CMM Medina J Brooks AJ. The growth hormone receptor: Mechanism of receptor activation, cell signaling, and physiological aspects. Front Endocrinol (Lausanne) (2018) 9:35. doi: 10.3389/fendo.2018.00035 29487568 PMC5816795

[80] Scheffler F Vandecandelaere A Soyez M Bosquet D Lefranc E Copin H. Follicular GH and IGF1 levels are associated with oocyte cohort quality: A pilot study. Front Endocrinol (Lausanne) (2021) 12:793621. doi: 10.3389/fendo.2021.793621 34925246 PMC8672194

[81] Velazquez MA Hadeler KG Herrmann D Kues WA Remy B Beckers JF. *In vivo* oocyte IGF-1 priming increases inner cell mass proliferation of *in vitro*-formed bovine blastocysts. Theriogenology (2012) 78(3):517–27. doi: 10.1016/j.theriogenology.2012.02.034 22538004

[82] Sato A Sarentonglaga B Ogata K Yamaguchi M Hara A Atchalalt K. Effects of insulin-like growth factor-1 on the *in vitro* maturation of canine oocytes. J Reprod Dev (2018) 64(1):83–8. doi: 10.1262/jrd.2017-145 PMC5830362 29212962

[83] Yu Y Yan J Li M Yan L Zhao Y Lian Y. Effects of combined epidermal growth factor, brain-derived neurotrophic factor and insulin-like growth factor-1 on human oocyte maturation and early fertilized and cloned embryo development. Hum Reprod (2012) 27(7):2146–59. doi: 10.1093/humrep/des099 22532606

[84] Brown-Borg HM Borg KE Meliska CJ Bartke A. Dwarf mice and the ageing process. Nature (1996) 384(6604):33. doi: 10.1038/384033a0 8900272

[85] Eicher EM Beamer WG. Inherited ateliotic dwarfism in mice. characteristics of the mutation, little, on chromosome 6. J Hered (1976) 67(2):87–91. doi: 10.1093/oxfordjournals.jhered.a108682 1270792

[86] Zhou Y Xu BC Maheshwari HG He L Reed M Lozykowski M. A mammalian model for laron syndrome produced by targeted disruption of the mouse growth hormone receptor/binding protein gene (the laron mouse). Proc Natl Acad Sci U.S.A. (1997) 94(24):13215–20. doi: 10.1073/pnas.94.24.13215 PMC24289 9371826

[87] Holzenberger M Dupont J Ducos B Leneuve P Geloen A Even PC. IGF-1 receptor regulates lifespan and resistance to oxidative stress in mice. Nature (2003) 421(6919):182–7. doi: 10.1038/nature01298 12483226

[88] Alcantara MR Salvatori R Alcantara PR Nobrega LM Campos VS Oliveira EC. Thyroid morphology and function in adults with untreated isolated growth hormone deficiency. J Clin Endocrinol Metab (2006) 91(3):860–4. doi: 10.1210/jc.2005-2555 16394080

[89] Laron Z Klinger B. Body fat in laron syndrome patients: effect of insulin-like growth factor I treatment. Horm Res (1993) 40(1-3):16–22. doi: 10.1159/000183762 8300045

[90] Oliveira JL Aguiar-Oliveira MH D'Oliveira A Jr. Pereira RM Oliveira CR Farias CT. Congenital growth hormone (GH) deficiency and atherosclerosis: effects of GH replacement in GH-naive adults. J Clin Endocrinol Metab (2007) 92(12):4664–70. doi: 10.1210/jc.2007-1636 17911170

[91] List EO Sackmann-Sala L Berryman DE Funk K Kelder B Gosney ES. Endocrine parameters and phenotypes of the growth hormone receptor gene disrupted (GHR-/-) mouse. Endocr Rev (2011) 32(3):356–86. doi: 10.1210/er.2010-0009 PMC3365798 21123740

[92] Dosouto C Calaf J Polo A Haahr T Humaidan P. Growth hormone and reproduction: Lessons learned from animal models and clinical trials. Front Endocrinol (Lausanne) (2019) 10:404. doi: 10.3389/fendo.2019.00404 31297089 PMC6607366

[93] Danilovich N Wernsing D Coschigano KT Kopchick JJ Bartke A. Deficits in female reproductive function in GH-R-KO mice; role of IGF-I. Endocrinology (1999) 140(6):2637–40. doi: 10.1210/endo.140.6.6992 10342852

[94] Baker J Hardy MP Zhou J Bondy C Lupu F Bellve AR. Effects of an Igf1 gene null mutation on mouse reproduction. Mol Endocrinol (1996) 10(7):903–18. doi: 10.1210/mend.10.7.8813730 8813730

[95] Shiomi-Sugaya N Komatsu K Wang J Yamashita M Kikkawa F Iwase A. Regulation of secondary follicle growth by theca cells and insulin-like growth factor 1. J Reprod Dev (2015) 61(3):161–8. doi: 10.1262/jrd.2014-107 PMC4498370 25740252

[96] Saccon TD Moreira F Cruz LA Mondadori RG Fang Y Barros CC. Ovarian aging and the activation of the primordial follicle reserve in the long-lived Ames dwarf and the short-lived bGH transgenic mice. Mol Cell Endocrinol (2017) 455:23–32. doi: 10.1016/j.mce.2016.10.015 27771355 PMC5397383

[97] Adams NR Briegel JR. Multiple effects of an additional growth hormone gene in adult sheep. J Anim Sci (2005) 83(8):1868–74. doi: 10.2527/2005.8381868x 16024706

[98] Ebner T Moser M Tews G. Is oocyte morphology prognostic of embryo developmental potential after ICSI? Reprod BioMed Online (2006) 12(4):507–12. doi: 10.1016/S1472-6483(10)62006-8 16740226

[99] Sousa M Cunha M Silva J Oliveira E Pinho MJ Almeida C. Ultrastructural and cytogenetic analyses of mature human oocyte dysmorphisms with respect to clinical outcomes. J Assist Reprod Genet (2016) 33(8):1041–57. doi: 10.1007/s10815-016-0739-8 PMC4974231 27221476

[100] Chandrashekar V Zaczek D Bartke A. The consequences of altered somatotropic system on reproduction. Biol Reprod (2004) 71(1):17–27. doi: 10.1095/biolreprod.103.027060 15028633

[101] Karamouti M Kollia P Kallitsaris A Vamvakopoulos N Kollios G Messinis IE. Growth hormone, insulin-like growth factor I, and leptin interaction in human cultured lutein granulosa cells steroidogenesis. Fertil Steril (2008) 90(4 Suppl):1444–50. doi: 10.1016/j.fertnstert.2007.08.076 18082739

[102] Pellegrini S Fuzzi B Pratesi S Mannelli M Criscuoli L Messeri G. In-vivo studies on ovarian insulin-like growth factor I concentrations in human preovulatory follicles and human ovarian circulation. Hum Reprod (1995) 10(6):1341–5. doi: 10.1093/HUMREP/10.6.1341 7593492

[103] Potashnik G Lunenfeld E Shwartz I Glezerman M Roberts CT Jr. LeRoith D. Endogenous plasma growth hormone and the occurrence of pregnancies in patients undergoing in-vitro fertilization and embryo transfer with ovarian stimulation. Hum Reprod (1995) 10(5):1065–9. doi: 10.1093/oxfordjournals.humrep.a136095 7657742

[104] Azari-Dolatabad N Raes A Pavani KC Asaadi A Angel-Velez D Van Damme P. Follicular fluid during individual oocyte maturation enhances cumulus expansion and improves embryo development and quality in a dose-specific manner. Theriogenology (2021) 166:38–45. doi: 10.1016/j.theriogenology.2021.02.016 33684781

[105] Mendoza C Ruiz-Requena E Ortega E Cremades N Martinez F Bernabeu R. Follicular fluid markers of oocyte developmental potential. Hum Reprod (2002) 17(4):1017–22. doi: 10.1093/humrep/17.4.1017 11925399

[106] Mendoza C Cremades N Ruiz-Requena E Martinez F Ortega E Bernabeu S. Relationship between fertilization results after intracytoplasmic sperm injection, and intrafollicular steroid, pituitary hormone and cytokine concentrations. Hum Reprod (1999) 14(3):628–35. doi: 10.1093/humrep/14.3.628 10221687

[107] Geisthoevel F Moretti-Rojas IM Rojas FJ Asch RH. Immunoreactive insulin-like growth factor I in human follicular fluid. Hum Reprod (1989) 4(1):35–8. doi: 10.1093/oxfordjournals.humrep.a136841 2708501

[108] Rabinovici J Dandekar P Angle MJ Rosenthal S Martin MC. Insulin-like growth factor I (IGF-I) levels in follicular fluid from human preovulatory follicles: correlation with serum IGF-I levels. Fertil Steril (1990) 54(3):428–33. doi: 10.1016/S0015-0282(16)53756-X 2118859

[109] Dakhly DMR Bassiouny YA Bayoumi YA Hassan MA Gouda HM Hassan AA. The addition of growth hormone adjuvant therapy to the long down regulation protocol in poor responders undergoing *in vitro* fertilization: Randomized control trial. Eur J Obstet Gynecol Reprod Biol (2018) 228:161–5. doi: 10.1016/j.ejogrb.2018.06.035 29957401

[110] Duffy JM Ahmad G Mohiyiddeen L Nardo LG Watson A. Growth hormone for in vitro fertilization. Cochrane Database Syst Rev (2010) 1):CD000099. doi: 10.1002/14651858.CD000099.pub3 PMC7058116 20091500

[111] Gomez E Tarin JJ Pellicer A. Oocyte maturation in humans: the role of gonadotropins and growth factors. Fertil Steril (1993) 60(1):40–6. doi: 10.1016/S0015-0282(16)56033-6 8513957

[112] Tu Z Ran H Zhang S Xia G Wang B Wang H. Molecular determinants of uterine receptivity. Int J Dev Biol (2014) 58(2-4):147–54. doi: 10.1387/ijdb.130345wh 25023680

[113] Bonilla-Musoles F Raga F Osborne NG Castillo JC Bonilla F Jr.. Endometrial receptivity: evaluation with ultrasound. Ultrasound Q (2013) 29(1):3–20. doi: 10.1097/RUQ.0b013e318281b60a 23435494

[114] Liu FT Wu Z Yan J Norman RJ Li R. The potential role of growth hormone on the endometrium in assisted reproductive technology. Front Endocrinol (Lausanne) (2020) 11:49. doi: 10.3389/fendo.2020.00049 32117072 PMC7033614

[115] Bondy CA Zhou J. Growth hormone, insulin-like growth factors and the female reproductive system. Adv Exp Med Biol (2005) 567:91–115. doi: 10.1007/0-387-26274-1_4 16370137

[116] Chilton BS Daniel JC Jr. Differences in the rabbit uterine response to progesterone as influenced by growth hormone or prolactin. J Reprod Fertil (1987) 79(2):581–7. doi: 10.1530/jrf.0.0790581 3572889

[117] Bezecny I Bartova J Skarda J. Growth hormone treatment increases oestrogen receptor concentration in the guinea-pig uterus. J Endocrinol (1992) 134(1):5–9. doi: 10.1677/joe.0.1340005 1380058

[118] Guzeloglu A Bilby TR Meikle A Kamimura S Kowalski A Michel F. Pregnancy and bovine somatotropin in nonlactating dairy cows: II. endometrial gene expression related to maintenance of pregnancy. J Dairy Sci (2004) 87(10):3268–79. doi: 10.3168/jds.S0022-0302(04)73463-3 15377606

[119] Santos JE Juchem SO Cerri RL Galvao KN Chebel RC Thatcher WW. Effect of bST and reproductive management on reproductive performance of Holstein dairy cows. J Dairy Sci (2004) 87(4):868–81. doi: 10.3168/jds.S0022-0302(04)73231-2 15259221

[120] Rutanen EM. Insulin-like growth factors in endometrial function. Gynecol Endocrinol (1998) 12(6):399–406. doi: 10.3109/09513599809012842 10065165

[121] Strowitzki T Wiedemann R Hepp H. Influence of growth factors EGF, IGF-1, and human growth hormone on human endometrial stromal cells in vitro. Ann N Y Acad Sci (1991) 626:308–11. doi: 10.1111/j.1749-6632.1991.tb37925.x 2058959

[122] Cui N Li AM Luo ZY Zhao ZM Xu YM Zhang J. Effects of growth hormone on pregnancy rates of patients with thin endometrium. J Endocrinol Invest (2019) 42(1):27–35. doi: 10.1007/s40618-018-0877-1 29671256

[123] Al-Ghamdi A Coskun S Al-Hassan S Al-Rejjal R Awartani K. The correlation between endometrial thickness and outcome of *in vitro* fertilization and embryo transfer (IVF-ET) outcome. Reprod Biol Endocrinol (2008) 6:37. doi: 10.1186/1477-7827-6-37 18764940 PMC2543019

[124] Wu Y Gao X Lu X Xi J Jiang S Sun Y. Endometrial thickness affects the outcome of *in vitro* fertilization and embryo transfer in normal responders after GnRH antagonist administration. Reprod Biol Endocrinol (2014) 12:96. doi: 10.1186/1477-7827-12-96 25296555 PMC4197319

[125] Zhao J Zhang Q Wang Y Li Y. Endometrial pattern, thickness and growth in predicting pregnancy outcome following 3319 IVF cycle. Reprod BioMed Online (2014) 29(3):291–8. doi: 10.1016/j.rbmo.2014.05.011 25070912

[126] Zhang T Li Z Ren X Huang B Zhu G Yang W. Endometrial thickness as a predictor of the reproductive outcomes in fresh and frozen embryo transfer cycles: A retrospective cohort study of 1512 IVF cycles with morphologically good-quality blastocyst. Med (Baltimore) (2018) 97(4):e9689. doi: 10.1097/MD.0000000000009689 PMC5794374 29369190

[127] Chen Y Liu F Nong Y Ruan J Guo Q Luo M. Clinical efficacy and mechanism of growth hormone action in patients experiencing repeat implantation failure. Can J Physiol Pharmacol (2018) 96(9):929–32. doi: 10.1139/cjpp-2017-0786 29726701

[128] Altmae S Mendoza-Tesarik R Mendoza C Mendoza N Cucinelli F Tesarik J. Effect of growth hormone on uterine receptivity in women with repeated implantation failure in an oocyte donation program: A randomized controlled trial. J Endocr Soc (2018) 2(1):96–105. doi: 10.1210/js.2017-00359 29379897 PMC5779111

[129] Xue-Mei W Hong J Wen-Xiang Z Yang L. The effects of growth hormone on clinical outcomes after frozen-thawed embryo transfer. Int J Gynaecol Obstet (2016) 133(3):347–50. doi: 10.1016/j.ijgo.2015.10.020 27101995

[130] Salle A Klein M Pascal-Vigneron V Dousset B Leclere J Weryha G. Successful pregnancy and birth after sequential cotreatment with growth hormone and gonadotropins in a woman with panhypopituitarism: a new treatment protocol. Fertil Steril (2000) 74(6):1248–50. doi: 10.1016/S0015-0282(00)01619-8 11119761

[131] Drakopoulos P Pluchino N Bischof P Cantero P Meyer P Chardonnens D. Effect of growth hormone on endometrial thickness and fertility outcome in the treatment of women with panhypopituitarism: A case report. J Reprod Med (2016) 61(1-2):78–82. 26995894

[132] Bassiouny YA Dakhly DMR Bayoumi YA Hashish NM. Does the addition of growth hormone to the *in vitro* fertilization/intracytoplasmic sperm injection antagonist protocol improve outcomes in poor responders? A randomized Controlled trial. Fertil Steril (2016) 105(3):697–702. doi: 10.1016/j.fertnstert.2015.11.026 26690008

[133] Eftekhar M Aflatoonian A Mohammadian F Eftekhar T. Adjuvant growth hormone therapy in antagonist protocol in poor responders undergoing assisted reproductive technology. Arch Gynecol Obstet (2013) 287(5):1017–21. doi: 10.1007/s00404-012-2655-1 23208461

[134] Du XF Yang XH Li J Hao M Guo YH. Growth hormone co-treatment within a GnRH agonist long protocol improves implantation and pregnancy rates in patients undergoing IVF-ET. Arch Gynecol Obstet (2016) 294(4):877–83. doi: 10.1007/s00404-016-4163-1 27488698

[135] Merhi Z Doswell A Krebs K Cipolla M. Vitamin d alters genes involved in follicular development and steroidogenesis in human cumulus granulosa cells. J Clin Endocrinol Metab (2014) 99(6):E1137–45. doi: 10.1210/jc.2013-4161 PMC4037738 24628555

[136] Gardner DK Schoolcraft WB. Culture and transfer of human blastocysts. Curr Opin Obstet Gynecol (1999) 11(3):307–11. doi: 10.1097/00001703-199906000-00013 10369209

[137] Homburg R West C Torresani T Jacobs HS. Cotreatment with human growth hormone and gonadotropins for induction of ovulation: a controlled clinical trial. Fertil Steril (1990) 53(2):254–60. doi: 10.1016/S0015-0282(16)53277-4 2105243

[138] Hart RJ Rombauts L Norman RJ. Growth hormone in IVF cycles: any hope? Curr Opin Obstet Gynecol (2017) 29(3):119–25. doi: 10.1097/GCO.0000000000000360 28306560

[139] Hart RJ. Use of growth hormone in the IVF treatment of women with poor ovarian reserve. Front Endocrinol (Lausanne) (2019) 10:500. doi: 10.3389/fendo.2019.00500 31396160 PMC6667844

[140] Tapanainen J Martikainen H Voutilainen R Orava M Ruokonen A Ronnberg L. Effect of growth hormone administration on human ovarian function and steroidogenic gene expression in granulosa-luteal cells. Fertil Steril (1992) 58(4):726–32. doi: 10.1016/S0015-0282(16)55319-9 1426317

[141] Younis JS Simon A Koren R Dorembus D Schenker JG Laufer N. The effect of growth hormone supplementation on *in vitro* fertilization outcome: a prospective randomized placebo-controlled double-blind study. Fertil Steril (1992) 58(3):575–80. doi: 10.1016/S0015-0282(16)55266-2 1387849

[142] Huang ZH Baxter RC Hughes SM Matson PL Lieberman BA Morris ID. Supplementary growth hormone treatment of women with poor ovarian response to exogenous gonadotrophins: changes in serum and follicular fluid insulin-like growth factor-1 (IGF-1) and IGF binding protein-3 (IGFBP-3). Hum Reprod (1993) 8(6):850–7. doi: 10.1093/oxfordjournals.humrep.a138153 7688379

[143] Homburg R Levy T Ben-Rafael Z. Adjuvant growth hormone for induction of ovulation with gonadotrophin-releasing hormone agonist and gonadotrophins in polycystic ovary syndrome: a randomized, double-blind, placebo controlled trial. Hum Reprod (1995) 10(10):2550–3. doi: 10.1093/oxfordjournals.humrep.a135743 8567768

[144] Oosterhuis GJ Vermes I Lambalk CB Michgelsen HW Schoemaker J. Insulin-like growth factor (IGF)-I and IGF binding protein-3 concentrations in fluid from human stimulated follicles. Hum Reprod (1998) 13(2):285–9. doi: 10.1093/humrep/13.2.285 9557823

[145] Homburg R Eshel A Abdalla HI Jacobs HS. Growth hormone facilitates ovulation induction by gonadotrophins. Clin Endocrinol (Oxf) (1988) 29(1):113–7. doi: 10.1111/j.1365-2265.1988.tb00252.x 3248352

[146] Spiliotis BE. Growth hormone insufficiency and its impact on ovarian function. Ann N Y Acad Sci (2003) 997:77–84. doi: 10.1196/annals.1290.009 14644812

[147] Park JK Murphy AA Bordeaux BL Dominguez CE Session DR. Ovulation induction in a poor responder with panhypopituitarism: a case report and review of the literature. Gynecol Endocrinol (2007) 23(2):82–6. doi: 10.1080/09513590601137533 17454157

[148] Kolibianakis EM Venetis CA Diedrich K Tarlatzis BC Griesinger G. Addition of growth hormone to gonadotrophins in ovarian stimulation of poor responders treated by in-vitro fertilization: a systematic review and meta-analysis. Hum Reprod Update (2009) 15(6):613–22. doi: 10.1093/humupd/dmp026 19561136

[149] Kyrou D Kolibianakis EM Venetis CA Papanikolaou EG Bontis J Tarlatzis BC. How to improve the probability of pregnancy in poor responders undergoing *in vitro* fertilization: a systematic review and meta-analysis. Fertil Steril (2009) 91(3):749–66. doi: 10.1016/j.fertnstert.2007.12.077 18639875

[150] Li XL Wang L Lv F Huang XM Wang LP Pan Y. The influence of different growth hormone addition protocols to poor ovarian responders on clinical outcomes in controlled ovary stimulation cycles: A systematic review and meta-analysis. Med (Baltimore) (2017) 96(12):e6443. doi: 10.1097/MD.0000000000006443 PMC5371493 28328856

[151] Yu X Ruan J He LP Hu W Xu Q Tang J. Efficacy of growth hormone supplementation with gonadotrophins *in vitro* fertilization for poor ovarian responders: an updated meta-analysis. Int J Clin Exp Med (2015) 8(4):4954–67. PMC4483949 26131068

[152] Zhang Y Zhang C Shu J Guo J Chang HM Leung PCK. Adjuvant treatment strategies in ovarian stimulation for poor responders undergoing IVF: a systematic review and network meta-analysis. Hum Reprod Update (2020) 26(2):247–63. doi: 10.1093/humupd/dmz046 32045470

[153] Harper K Proctor M Hughes E. Growth hormone for in vitro fertilization. Cochrane Database Syst Rev (2003) 3):CD000099. doi: 10.1002/14651858.CD000099 12917883

[154] Zhu J Wang Y Chen L Liu P Li R Qiao J. Growth hormone supplementation may not improve live birth rate in poor responders. Front Endocrinol (Lausanne) (2020) 11:1. doi: 10.3389/fendo.2020.00001 32038495 PMC6990136

[155] Oron G Son WY Buckett W Tulandi T Holzer H. The association between embryo quality and perinatal outcome of singletons born after single embryo transfers: a pilot study. Hum Reprod (2014) 29(7):1444–51. doi: 10.1093/humrep/deu079 24812313

[156] Zhu J Lian Y Li M Chen L Liu P Qiao J. Does IVF cleavage stage embryo quality affect pregnancy complications and neonatal outcomes in singleton gestations after double embryo transfers? J Assist Reprod Genet (2014) 31(12):1635–41. doi: 10.1007/s10815-014-0351-8 PMC4250469 25326318

[157] Gosden RG. Oogenesis as a foundation for embryogenesis. Mol Cell Endocrinol (2002) 186(2):149–53. doi: 10.1016/S0303-7207(01)00683-9 11900888

[158] Swain JE Pool TB. ART failure: oocyte contributions to unsuccessful fertilization. Hum Reprod Update (2008) 14(5):431–46. doi: 10.1093/humupd/dmn025 18603645

[159] Izadyar F Zhao J Van Tol HT Colenbrander B Bevers MM. Messenger RNA expression and protein localization of growth hormone in bovine ovarian tissue and in cumulus oocyte complexes (COCs) during *in vitro* maturation. Mol Reprod Dev (1999) 53(4):398–406. doi: 10.1002/(SICI)1098-2795(199908)53:4<398::AID-MRD5>3.0.CO;2-I 10398415

[160] Li J Chen Q Wang J Huang G Ye H. Does growth hormone supplementation improve oocyte competence and IVF outcomes in patients with poor embryonic development? A randomized Controlled trial. BMC Pregnancy Childbirth (2020) 20(1):310. doi: 10.1186/s12884-020-03004-9 32434490 PMC7238549

[161] Ojosnegros S Seriola A Godeau AL Veiga A. Embryo implantation in the laboratory: an update on current techniques. Hum Reprod Update (2021) 27(3):501–30. doi: 10.1093/humupd/dmaa054 33410481

[162] Macklon NS Stouffer RL Giudice LC Fauser BC. The science behind 25 years of ovarian stimulation for *in vitro* fertilization. Endocr Rev (2006) 27(2):170–207. doi: 10.1210/er.2005-0015 16434510

[163] Moreira F Badinga L Burnley C Thatcher WW. Bovine somatotropin increases embryonic development in superovulated cows and improves post-transfer pregnancy rates when given to lactating recipient cows. Theriogenology (2002) 57(4):1371–87. doi: 10.1016/S0093-691X(01)00719-1 12013456

[164] Cakmak H Taylor HS. Implantation failure: molecular mechanisms and clinical treatment. Hum Reprod Update (2011) 17(2):242–53. doi: 10.1093/humupd/dmq037 PMC3039220 20729534

[165] Swerdlow AJ Cooke R Beckers D Borgstrom B Butler G Carel JC. Cancer risks in patients treated with growth hormone in childhood: The SAGhE European cohort study. J Clin Endocrinol Metab (2017) 102(5):1661–72. doi: 10.1210/jc.2016-2046 PMC6061931 28187225

[166] Molitch ME Clemmons DR Malozowski S Merriam GR Vance ML Endocrine S. Evaluation and treatment of adult growth hormone deficiency: an endocrine society clinical practice guideline. J Clin Endocrinol Metab (2011) 96(6):1587–609. doi: 10.1210/jc.2011-0179 21602453

[167] Jeffcoate W. Growth hormone therapy and its relationship to insulin resistance, glucose intolerance and diabetes mellitus: a review of recent evidence. Drug Saf (2002) 25(3):199–212. doi: 10.2165/00002018-200225030-00005 11945115

[168] Ranke MB. Effects of growth hormone on the metabolism of lipids and water and their potential in causing adverse events during growth hormone treatment. Horm Res (1993) 39(3-4):104–6. doi: 10.1159/000182707 8262469

[169] van Bunderen CC van Varsseveld NC Erfurth EM Ket JC Drent ML. Efficacy and safety of growth hormone treatment in adults with growth hormone deficiency: a systematic review of studies on morbidity. Clin Endocrinol (Oxf) (2014) 81(1):1–14. doi: 10.1111/cen.12477 24750271

